# High dietary calcium to phosphorus ratio is associated with high prevalence of kidney stone

**DOI:** 10.1097/MD.0000000000040778

**Published:** 2024-12-13

**Authors:** Weiyu Zhang, Benxing Lou, Yu Peng, Feng Wu, Dan Zhang, Qi Wang

**Affiliations:** a Department of Urology, Peking University People’s Hospital, Beijing, China; b The Institute of Applied Lithotripsy Technology, Peking University, Beijing, China; c Department of Urology, Tongliao Second People’s Hospital, Tongliao, Inner Mongolia; d Naval Aviation University, Yantai, Shandong Province, China; e Institute for Disease Control and Prevention, Chinese PLA, Beijing, China; f Zigong Affiliated Hospital of Southwest Medical University, Zigong, Sichuan, China.

**Keywords:** calcium to phosphorus ratios, kidney stones, NHANS, risk

## Abstract

Kidney stones formation is a multifactorial condition and influenced, in some degree, by dietary habits. Authoritative clinical guidelines published nutritional recommendations for individuals prone to kidney stone formation. The association between dietary intake calcium to phosphorus (Ca/P) ratios and the prevalence of kidney stones is not well-established in extensive epidemiological studies. Data from the National Health and Nutrition Examination Survey 2017 to 2018 database were utilized in this study. A total of 3 149 participants with a history of kidney stones were enrolled in the present analysis. The participants were categorized into 4 groups based on their dietary Ca/P ratio, divided by quartiles, with quartile 1 representing the lowest ratio and quartile 4 indicating the highest ratio. We applied survey-weighting to all the data and conducted logistic regression models to assess the connections between Ca/P ratio and the likelihood of developing kidney stones. We utilized restricted cubic spline analysis to assess the nonlinear relationship between dietary Ca/P ratio intake and the risk of kidney stones. In a fully adjusted model referred by quartile 1, participants in quartile 4 had a significantly higher rate of kidney stones (odds ratio 1.5697, *P* < .001). Compared with single calcium or phosphorus consumption, the Ca/P ratio was found to be a better predictor of the risk of kidney stones. The quartile analysis suggested an appropriate Ca/P ratio of 0.5513 to 0.6810 to meet a lower risk of kidney stones. There was a significant association between dietary Ca/P ratio intake and the risk of kidney stones. A moderate intake of dietary Ca/P ratio was recommended.

## 1. Introduction

Kidney stones are mineral deposits that form in the renal pelvis due to urine with an exceptionally high level of saturation. The prevalence of kidney stones varies among different races and geographic regions, significantly influenced by diet, lifestyle, and metabolism.^[1]^ The relationship between diet and kidney stone formation is well-known and widely acknowledged. Major clinical guidelines^[2–4]^ recommend prioritizing dietary and lifestyle adjustments. These include increasing water intake to promote adequate diuresis, reducing consumption of meat and poultry, minimizing salt intake, avoiding overeating and sugary beverages, increasing vegetable consumption, and maintaining a balanced intake of essential microelements.

Worldwide, about 80% of kidney stones consist primarily of calcium (Ca) compounds, with calcium oxalate and calcium phosphate being the main constituents.^[5]^ Individuals with kidney stone history are recommended consumption of dietary Ca at 1000 to 1200 mg/day.^[2–4]^ Abnormal, too high or too low, dietary Ca intake leading to urinary stones is well-known to all.^[6,7]^ High phosphorus (P) intake is associated with a positive P balance, which correlates with renal calcification.^[8]^ But it has not been confirmed in a wide range of studies. Inappropriate P intake can disrupt Ca–P metabolism and increase the risk of kidney stones. This imbalance can result in hyperphosphatemia and hypercalciuria, both of which are key factors in kidney stone development.^[9]^ Maintaining a balanced dietary Ca and P intake, particularly with an appropriate Ca/P ratio, is important to lower the risk of kidney stones and maintain optimal mineral metabolism.^[10,11]^ Some recent studies have explored P’s impact on kidney health and highlighted that an imbalanced Ca/P ratio may contribute to renal calcification and dysfunction. However, comprehensive investigations focusing on how variations in the Ca/P ratio influence kidney stone formation are still scarce.

The National Health and Nutrition Examination Survey (NHANES) is a highly representative dataset used extensively in nutrition research.^[12]^ It collects health and nutritional data through a combination of interviews, physical exams, and laboratory tests, representing the noninstitutionalized U.S. population. In the present study, we aim to analyze the relationship between the dietary Ca/P ratio and the prevalence of kidney stones using NHANES data. By examining the dietary patterns of a representative U.S. population, we seek to determine whether variations in the Ca/P ratio are associated with differences in kidney stone risk.

Looking forward, our study’s findings have the potential to influence public health strategies, particularly in dietary guidelines for kidney stone prevention. These results could pave the way for more personalized dietary interventions, especially for high-risk individuals, offering a clearer path to reducing kidney stone formation.

## 2. Methods

### 2.1. Study design and participants

The NHANES is a set of comprehensive, multi-stage cross-sectional surveys performed by the Center for Disease Control and Prevention. These surveys are aimed at assessing the health and nutritional profiles of adults and children throughout the United States, using nationally representative samples. Researchers have delved into the NHANES database to uncover the causes, comprehend the epidemiology, and identify new biomarkers for various diseases. The methods and data collection procedure of NHANES are described in in the NHANES website (http://www.cdc.gov/nchs/nhanes.htm). Given the low prevalence of these conditions in the population, patients with specific diseases, such as renal insufficiency and metabolic disorders, were not excluded. Ethical approval was not required for analysis of these deidentified open-source data.

**Figure 1.**
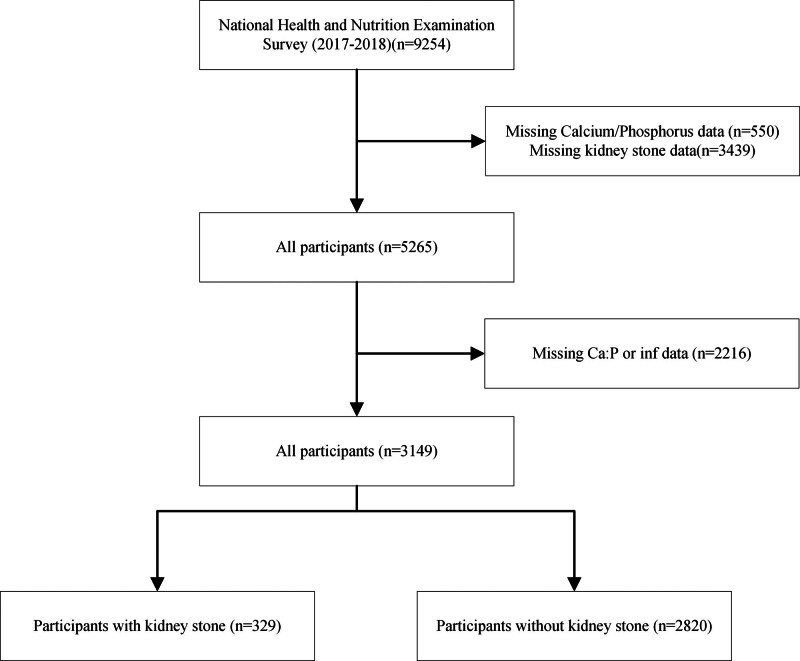
Research flowchart.

This study examining Ca and P dietary intake, using data from the 2017 to 2018 NHANES cycle, relied on information provided by the National Center for Health Statistics, a division of the Center for Disease Control and Prevention.^[13]^ Inclusion criteria: participants with a history of kidney stones, individuals with available dietary intake data for Ca and P. Exclusion criteria: participants with missing values for Ca and P intakes, individuals who reported zero intake of Ca and P, participants with specific diseases such as renal insufficiency and metabolic disorders were not excluded due to their low prevalence in the population. Out of 9254 eligible participants, only 3149 individuals were included in the analysis after applying the inclusion and exclusion criteria. The participant flowchart was shown in Figure 1.

### 2.2. Assessment of dietary data

We computed Ca/P ratio by using mass units for both Ca and P which was released in elemental form, based on the NHANES dietary intake and supplement information. Data on Ca and P intake is from both dietary sources and supplemental sources. The intake information is collected through 24-hour dietary recall interviews and a dietary supplement questionnaire, which provide comprehensive data on total nutrient intake from both regular food consumption and the use of supplements. This allows for a more complete assessment of total Ca and P intake and its potential health impacts. Participants were divided into 4 groups according to the quartiles of their dietary Ca/P ratio, with quartile 1 representing the lowest ratio and quartile 4 indicating the highest ratio. NHANES collected dietary information through questionnaires, capturing details on the frequency, type, and quantity of supplements taken in the previous 30 days, which were then used to compute averages. Additionally, dietary consumption data were obtained through two 24-hour recalls, one conducted in the mobile examination centers and the other via telephone interviews. Data on nutrients and food items including total energy (kcal), total water drink (g), alcohol (gm), vitamin B6 (mg), vitamin C (mg), vitamin D (mcg), magnesium (mg), Sodium (mg), Potassium (mg), Ca (mg), P (mg), and caffeine intake (mg/day) was obtained from the total nutrient file, which contains summed nutrients for an individual from all foods and beverages provided on the dietary recall.

### 2.3. Assessment of covariates

The diagnosis of kidney stones was from the questionnaire data about kidney conditions. Demographic information such as age, race/ethnicity, education levels, marital status, and the ratio of family income to the poverty level were gathered from the demographic questionnaires. Body mass index (BMI) data was obtained from physical examination records. Information on vigorous/moderate recreational activities, total water consumption, alcohol intake, and smoking status was extracted from the health questionnaires. Race/ethnicity was categorized into 4 groups: Mexican American, non-Hispanic White, non-Hispanic Black, and other racial backgrounds. Education was categorized into 5 levels: <9th grade, 9 to 11th grade, high school graduate, some college or AA degree, and college graduate or above. Income ratio to poverty was divided into <1, 1 to 5, or >5. The data resource was displayed in Table S1, Supplemental Digital Content, http://links.lww.com/MD/O172.

### 2.4. Statistical analysis

For the primary analysis of nutrient intake distribution for Ca, P, and Ca/P ratios, we employed the procedures established by the National Cancer Institute. These procedures involve a series of macros, which are built on nonlinear mixed models, designed to estimate the typical intake distribution of the population based on 24-hour dietary recall data. We conducted a secondary analysis for the study participants using the R SURVEYMEANS procedure. The use of the DOMAIN statement allowed us to generate estimates for subpopulations categorized by age (ranging 20–80) and gender (male and female). This method is primarily rooted in the Taylor expansion, as it relies on sampling error estimates derived from the intricate sample design. Due to the intricate sampling approach employed by NHANES in collecting survey data, weights were applied to address both bias (nonresponse) and the uneven likelihood of sample selection. All analyses were conducted using R version 4.3.0, with stratum adjustment for the NHANES center incorporated in the estimation process.

We compared baseline characteristics among individuals with and without kidney stone based on independent *t* tests, and chi-square test. We employed weighted logistic regression to compute odds ratios (ORs) and their corresponding 95% confidence intervals to assess the risk of recurrent kidney stones within each Ca/P ratio quartile. We derived 3 separate logistic regression models. Model 1 was a crude model. Model 2 was adjusted for age and sex. Model 3 was fully adjusted for age, BMI, gender, race, family income, educational level, marital status, vigorous and moderate recreational physical activity, smoking, energy, total water drank, alcohol, total dietary, and supplements intakes. We applied restricted cubic spline analysis with 3 piecewise points to assess the nonlinear relationships between the dietary Ca/P ratio intake and the risk of kidney stones.

For continuous variables, we have reported the mean value along with the standard deviation. For categorical variables, we have presented the frequencies or percentages. A significance level of *P* < .05 was adopted.

## 3. Results

### 3.1. Baseline characteristics of participants

The current study included a cohort of 3149 participants sourced from the NHANES database. The baseline characteristics of participants in this study are provided for the entire cohort and stratified by quartiles of Ca/P ratio in dietary intake (Table 1). The average Ca/P ratio for quartiles 1 to 4 were 0.45 ± 0.08, 0.62 ± 0.04, 0.74 ± 0.04, 0.97 ± 0.15, respectively. The average age of participants was 52.02 years, with the highest mean age in Quartile 1 (53.16 years) and the lowest in Quartile 4 (50.64 years). The proportion of men was 45.82% overall, with a decreasing trend from Quartile 1 (53.43%) to Quartile 4 (37.61%). The weighted prevalence of kidney stones was 10.45% and the overall Ca/P ratio was 0.70 ± 0.21. There is a significant difference between these quartiles (*P* < .05), with Quartile 4 showing the highest rate (12.07%) and Quartile 2 the lowest (8.39%).

**Table 1:** Baseline characteristics of participants.

	Overall	Calcium-to-phosphorus ratio in diet intake				*P* value
		Quartile 1 (n = 788)	Quartile 2 (n = 787)	Quartile 3 (n = 787)	Quartile 4 (n = 787)	
Calcium-to-phosphorus ratio [M (SD)]	0.70 (0.21)	0.45 (0.08)	0.62 (0.04)	0.74 (0.04)	0.97 (0.15)	.000***
Rate of kidney stones (%)	329 (10.45)	77 (9.77)	66 (8.39)	91 (11.56)	95 (12.07)	.039*
Men (%)	1443 (45.82)	421 (53.43)	373 (47.4)	353 (44.85)	296 (37.61)	.000***
Age [years, M (SD)]	52.02 (17.15)	53.16 (16.60)	51.91 (17.11)	52.37 (17.33)	50.64 (17.48)	.0095**
*Race (%*)						
Mexican American	421 (13.37)	97 (12.31)	116 (14.74)	101 (12.83)	107 (13.60)	.720
Other Hispanic	307 (9.75)	69 (8.76)	62 (7.88)	92 (11.69)	84 (10.67)	.043*
Non-Hispanic White	1168 (37.09)	227 (28.81)	283 (35.96)	331 (42.06)	327 (41.55)	.000***
Non-Hispanic Black	1109 (35.22)	353 (44.80)	299 (37.99)	225 (28.59)	232 (29.48)	.000***
Other race	144 (4.57)	42 (5.33)	27 (3.43)	38 (4.83)	37 (4.70)	.880
*Education (%*)						
<9th grade	223 (7.08)	55 (6.98)	53 (6.73)	69 (8.77)	46 (5.84)	.740
9–11th grade	299 (9.50)	71 (9.01)	77 (9.78)	71 (9.02)	80 (10.17)	.560
High school graduate	695 (22.07)	144 (18.27)	167 (21.22)	174 (22.11)	210 (26.68)	.000***
Some college or AA degree	1062 (33.72)	292 (37.06)	255 (32.40)	243 (30.88)	272 (34.56)	.230
College graduate or above	870 (27.63)	226 (28.68)	235 (29.86)	230 (29.22)	179 (22.74)	.0097**
Married (%)	1669 (53.00)	448 (56.85)	449 (57.05)	412 (52.35)	360 (45.74)	.000***
*Ratio of family income to poverty (%*)						
<1	779 (24.74)	179 (22.72)	183 (23.25)	206 (26.18)	211 (26.81)	.027*
1–5	1789 (56.81)	459 (58.25)	434 (55.15)	440 (55.91)	456 (57.94)	.980
>5	581 (18.45)	150 (19.04)	170 (21.60)	141 (17.92)	120 (15.25)	.015*
*BMI (%*)						
<20	118 (3.75)	28 (3.55)	24 (3.05)	28 (3.56)	38 (4.83)	.150
20–25	636 (20.20)	158 (20.05)	167 (21.22)	159 (20.20)	152 (19.31)	.610
25–30	1051 (33.38)	287 (36.42)	246 (31.26)	260 (33.04)	258 (32.78)	.220
>30	1344 (42.68)	315 (39.97)	350 (44.47)	340 (43.20)	339 (43.07)	.310
Vigorous/moderate recreational activities for at least 10 min continuously per week (%)	1551 (49.25)	350 (44.42)	398 (50.57)	414 (52.60)	389 (49.43)	.032*
Smoked at least 100 cigarettes in life (%)	1315 (41.76)	342 (43.40)	325 (41.30)	310 (39.39)	338 (42.95)	.680
*Daily intake [M (SD)]*						
Total energy (kcal)	1996.39 (767.47)	2055.80 (770.20)	2016.00 (733.70)	2046.50 (787.46)	1867.20 (763.87)	.000***
Total water drank (g)	2506.65 (2057.40)	2042.40 (1592.10)	2371.80 (1761.10)	2563.30 (2122.00)	3049.70 (2505.60)	.000***
Alcohol (gm)	13.59 (34.82)	17.94 (44.44)	14.43 (33.64)	13.04 (32.74)	8.92 (25.18)	.000***
Vitamin B6 (mg)	4.07 (2.80)	4.28 (2.60)	4.12 (3.09)	3.97 (2.16)	3.92 (3.20)	.0062**
Vitamin C (mg)	157.48 (142.92)	148.57 (134.21)	151.76 (132.78)	161.70 (137.52)	167.88 (164.30)	.0029**
Vitamin D (μg)	8.58 (8.70)	7.69 (10.33)	8.03 (7.49)	8.95 (8.77)	9.64 (7.79)	.000***
Magnesium (mg)	588.45 (247.87)	593.99 (250.27)	587.56 (245.99)	604.36 (250.46)	567.88 (243.73)	.120
Sodium (mg)	6525.71 (2692.01)	6933.60 (2912.60)	6669.20 (2612.10)	6594.80 (2658.80)	5904.70 (2461.70)	.000***
Potassium (mg)	5083.64 (2059.33)	5190.90 (2030.40)	5169.20 (2038.80)	5197.40 (2088.90)	4776.90 (2052.00)	.0002***
Caffeine (mg)	279.58 (286.16)	288.66 (273.42)	285.47 (261.42)	276.18 (281.46)	267.99 (324.49)	.120

BMI = body mass index, SD = standard deviation.

* *P* < .05.

** *P* < .01.

*** *P* < .001.

In terms of racial distribution, the largest group was non-Hispanic White (37.09%), followed by non-Hispanic Black (35.22%), with Quartile 1 having a higher proportion of non-Hispanic Blacks (44.80%) and Quartile 3 having the highest proportion of non-Hispanic Whites (42.06%). Education levels also varied, with 33.72% having some college education or an AA degree and 27.63% being college graduates or above. A significant number of participants (53.00%) were married, with the highest percentage in Quartile 2 (57.05%). The study also provided data on participants’ BMI, showing that 42.68% had a BMI over 30. Additionally, 49.25% reported engaging in recreational physical activities, and 41.76% had smoked at least 100 cigarettes in their lifetime.

### 3.2. Difference in consumption of dietary Ca/P ratio in subgroup analyses

In the subgroup analyses of the study on the Ca/P ratio in diet and kidney stone prevalence, the dietary Ca/P ratio exhibited significant variability across different groups (Table 1). The overall prevalence of kidney stones was observed to increase with higher dietary Ca/P ratios. Specifically, the highest quartile (Q4) of dietary Ca/P ratio (0.97 ± 0.15) had a higher prevalence of kidney stones (12.07%) compared to the lower quartiles, with the lowest prevalence seen in the second quartile (Q2) at 8.39%.

Gender differences were apparent, with men showing a higher prevalence of kidney stones in Quartile 4 (14.53%) compared to Quartile 1 (12.11%), though this increase was not statistically significant. However, among women, the increase was more pronounced and statistically significant, rising from 5.80% in Quartile 2 to 10.59% in Quartile 4 (*P* = .0426). Age also played a role in the association between the Ca/P ratio and kidney stone prevalence. Younger individuals (≤30 years) showed a lower prevalence, while participants aged 31 to 40 demonstrated a significant increase in kidney stone prevalence in the higher quartiles, particularly Quartile 4 (16.30%, *P* = .0025). Other subgroup analyses based on race, education, and physical activity indicated some trends, but the statistical significance varied. For instance, those who engaged in less physical activity and smoked at least 100 cigarettes in their lifetime were more likely to experience higher kidney stone prevalence with increasing Ca/P ratio, showing statistical significance (*P* < .05).

These findings highlight the complex interaction between dietary Ca/P ratios and demographic factors in the prevalence of kidney stones.

### 3.3. Association of dietary intake Ca/P ratio and the risk of kidney stones

**Figure 2.**
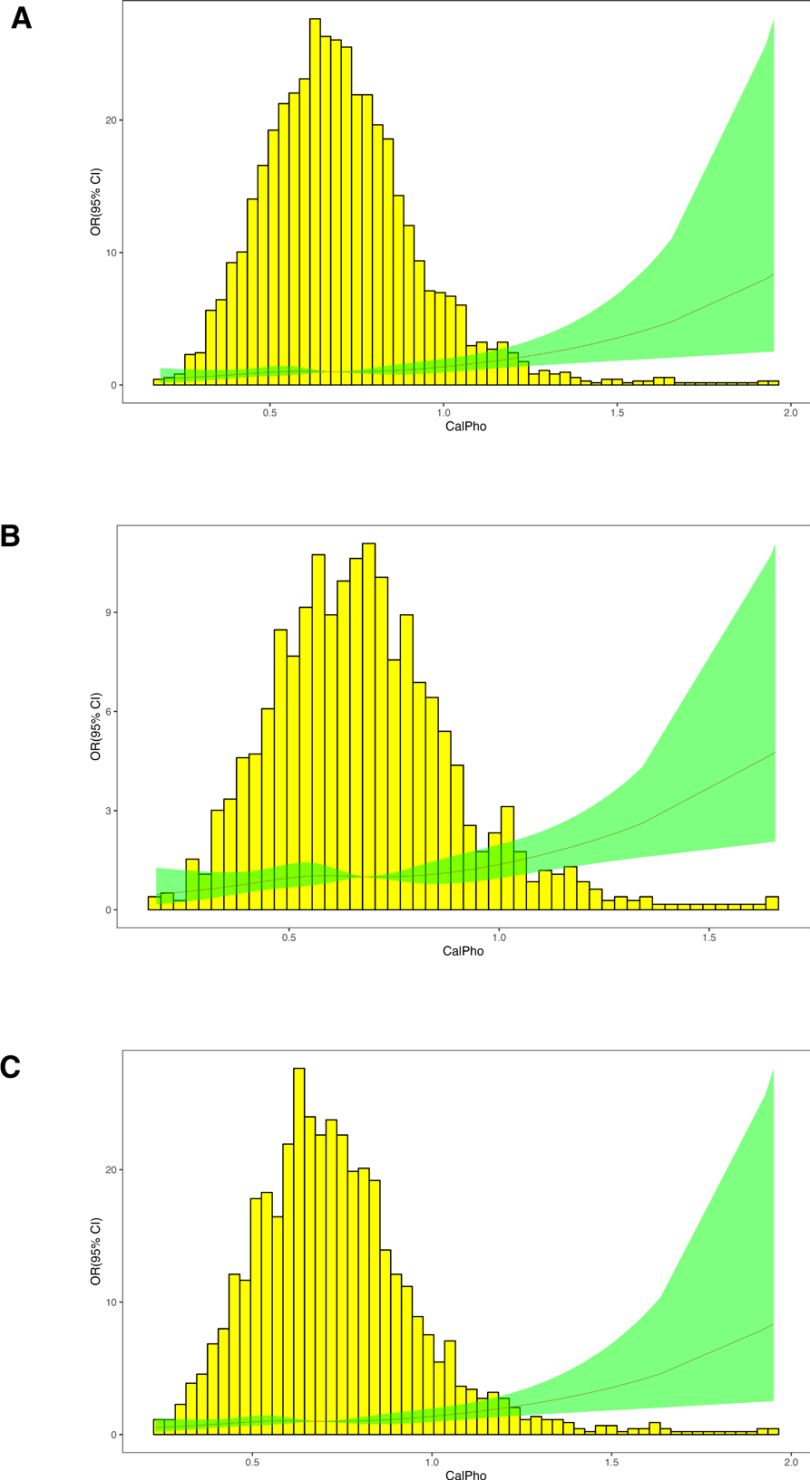
RCS analysis on the association between the dietary intake Ca/P ratio and the risk of kidney stones. (A) RCS curve of the association between dietary intake Ca/P ratio and kidney stones among all the participants. (B) RCS curve of the association between dietary intake Ca/P ratio and kidney stones among male participants. (C) RCS curve of the association between dietary intake Ca/P ratio and kidney stones among female participants. RCS = restricted cubic spline.

The association between dietary Ca/P ratio and the risk of kidney stones is a key focus of this study. The OR serves as an indicator of the intensity of the link between dietary Ca/P ratio intake and the occurrence of kidney stones, as demonstrated in Table 2. Compared to participants in Quartile 1, those in Quartile 4 had a significantly higher risk of developing kidney stones. The adjusted OR for Quartile 4, after accounting for potential confounding factors such as age, gender, BMI, educational level, and other dietary and lifestyle variables, was 1.57 (95% confidence interval: 1.11–2.22; *P* = .011), indicating a strong positive association between a higher dietary Ca/P ratio and kidney stone formation. Restricted cubic spline analysis was employed to support this result. Figure 2A shows the distribution of the Ca/P ratio among all the participants. As the Ca/P ratio increases in women (Fig. 2C), the OR of kidney stones increases faster than in men (Fig. 2B).

**Table 2:** Association between intake calcium-to-phosphorus ratio and kidney stones.

Ca–P ratio	OR (95% CI), *P*-value		
	Model 1	Model 2	Model 3
Quartile 1 <0.5513	1.0000 (reference)	1.0000 (reference)	1.0000 (reference)
Quartile 2 0.5513~0.6810	0.8113 (0.4651,1.4150) .461	0.8919 (0.5086,1.5638) .690	0.8365 (0.4733,1.4784) .539
Quartile 3 0.6810~0.8172	1.3665 (0.8894,2.0997) .154	1.4956 (0.9680,2.3110) .070	1.4298 (0.9147,2.2348) .117
Quartile 4 0.8172>	1.4003 (1.0211,1.9202) .037*	1.6372 (1.1842,2.2633) .003**	1.5697 (1.1099,2.2198) .011*
*P* value	.0054**	.0000***	.0000***

Data are presented as OR (95% CI).

Model 1: crude model.

Model 2: adjusted for age and sex.

Model 3: adjusted for age, gender, BMI, educational level, family income, marital status, race, smoking, vigorous and moderate recreational physical activity, total water drank, energy, alcohol, total intakes (from dietary and supplements) vitamins B6, vitamins C, vitamins D, caffeine, magnesium, sodium, and potassium.

BMI = body mass index, CI = confidence interval, OR = odds ratio.

* *P* < .05.

** *P* < .01.

*** *P* < .001.

Subgroup analyses highlighted that this association was more pronounced in specific groups, particularly women. Women in Quartile 4 exhibited a higher prevalence of kidney stones (10.59%) compared to those in Quartile 2 (5.80%), with a statistically significant difference (*P* = .0426). Additionally, age appeared to influence the risk, with those in the 31 to 40 age group showing the strongest association between Ca/P ratio and kidney stone prevalence (*P* = .0025).

We further analyzed the impact of Ca and P individually as influencing factors on the occurrence of kidney stones (Table S2, Supplemental Digital Content, http://links.lww.com/MD/O172). For Ca intake, the OR range from 1.0004 to 1.2796 across the models, but none of the results are statistically significant, with *P*-values above .05 (.926, .997, and .160). This suggests no significant association between Ca intake and kidney stone prevalence. In contrast, P intake shows a potentially protective effect in Model 2, which adjusts for age and sex (OR = 0.7002, *P* = .011). This indicates that higher P intake might be linked to a reduced risk of kidney stones in this specific model. However, the association is not statistically significant in Model 1 (crude) and Model 3 (fully adjusted for various lifestyle factors), with *P*-values of .086 and .078, respectively. As an influencing factor, the Ca/P shows a stronger correlation with the occurrence of kidney stones than Ca or P alone. This indicates that in dietary adjustments, focusing solely on Ca or P intake is insufficient.

The result highlights a positive association between high dietary Ca/P ratios and an increased risk of kidney stones, particularly in specific demographic subgroups, supporting the need for dietary adjustments in populations at risk.

### 3.4. Subgroup analysis of dietary intake Ca/P ratio and the risk of kidney stones

To investigate how various demographic and lifestyle factors impact on prevalence of kidney stone, a subgroup analysis was performed (Table 3).

**Table 3:** Subgroup analysis of dietary intake Ca/P ratio and kidney stones.

	Overall prevalence	Calcium-to-phosphorus ratio in dietary intake				*P* value
		Quartile 1 (n = 788)	Quartile 2 (n = 787)	Quartile 3 (n = 787)	Quartile 4 (n = 787)	
Prevalence of kidney stones (%)	329 (10.45)	77 (9.77)	66 (8.39)	91 (11.56)	95 (12.07)	.0651*
*Gender (prevalence, 95% CI*)						
Male	186 (12.89)	51 (12.11)	42 (11.26)	50 (14.16)	43 (14.53)	<.001***
Female	143 (8.38)	26 (7.08)	24 (5.80)	41 (9.45)	52 (10.59)	
*Age (prevalence, 95% CI*)						
≤30	16 (3.62)	7 (7.61)	3 (2.56)	4 (3.77)	2 (1.57)	.005**
31–40	45 (9.24)	3 (2.78)	8 (6.84)	12 (9.45)	22 (16.30)	
41–50	53 (10.95)	16 (12.40)	14 (11.76)	10 (8.85)	13 (10.57)	
51–60	65 (11.15)	16 (9.82)	13 (9.22)	16 (11.35)	20 (14.49)	
61–70	79 (12.42)	18 (11.32)	14 (8.38)	25 (15.53)	22 (14.77)	
>70	71 (13.73)	17 (12.41)	14 (11.11)	24 (17.27)	16 (13.91)	
*Race (prevalence, 95% CI*)						
Mexican American	34 (8.08)	6 (6.19)	5 (4.31)	13 (12.87)	10 (9.35)	<.001***
Other Hispanic	30 (9.77)	7 (10.14)	5 (8.06)	12 (13.04)	6 (7.14)	
Non-Hispanic White	167 (14.30)	33 (14.54)	30 (10.60)	47 (14.20)	57 (17.43)	
Non-Hispanic Black	81 (7.30)	29 (8.22)	23 (7.69)	13 (5.78)	16 (6.90)	
Other race	17 (11.81)	2 (4.76)	3 (11.11)	6 (15.79)	6 (16.22)	
*Education (prevalence, 95% CI*)						
<9th grade	15 (6.73)	5 (9.09)	1 (1.89)	6 (8.70)	3 (6.52)	.015*
9–11th grade	32 (10.70)	5 (7.04)	7 (9.09)	10 (14.08)	10 (12.50)	
High school graduate	68 (9.78)	12 (8.33)	11 (6.59)	15 (8.62)	30 (14.29)	
Some college or AA degree	140 (13.18)	37 (12.67)	32 (12.55)	38 (15.64)	33 (12.13)	
College graduate or above	74 (8.51)	18 (7.96)	15 (6.38)	22 (9.57)	19 (10.61)	
*Marriage (prevalence, 95% CI*)						
Married	191 (11.44)	46 (10.27)	41 (9.13)	59 (14.32)	45 (12.50)	<.001***
Single	138 (9.32)	31 (9.12)	25 (7.40)	32 (8.53)	50 (11.71)	
*Ratio of family income to poverty (prevalence, 95% CI*)						
<1	73 (9.37)	20 (11.17)	9 (4.92)	22 (10.68)	22 (10.43)	.004**
1–5	192 (10.73)	40 (8.71)	45 (10.37)	52 (11.82)	55 (12.06)	
>5	64 (11.02)	17 (11.33)	12 (7.06)	17 (12.06)	18 (15.00)	
*BMI (prevalence, 95% CI*)						
<20	11 (9.32)	4 (14.29)	1 (4.17)	3 (10.71)	3 (7.89)	.827
20–25	40 (6.29)	7 (4.43)	8 (4.79)	9 (5.66)	16 (10.53)	
25–30	106 (10.09)	32 (11.15)	18 (7.32)	32 (12.31)	24 (9.30)	
>30	172 (12.80)	34 (10.79)	39 (11.14)	47 (13.82)	52 (15.34)	
*Vigorous/moderate recreational activities for at least 10 min continuously per week (prevalence, 95% CI*)						
Yes	161 (10.38)	39 (11.14)	36 (9.05)	43 (10.39)	43 (11.05)	.032*
No	168 (10.51)	38 (8.68)	30 (7.71)	48 (12.87)	52 (13.07)	
*Smoked at least 100 cigarettes in life (prevalence, 95% CI*)						
Yes	166 (12.62)	35 (10.23)	32 (9.85)	47 (15.16)	52 (15.38)	.678
No	163 (8.89)	42 (9.42)	34 (7.36)	44 (9.22)	43 (9.58)	

BMI = body mass index, CI = confidence interval.

* *P* < .05.

** *P* < .01.

*** *P* < .001.

Gender-specific analysis revealed significant differences among females (*P* = .0426), where the prevalence increased from 7.08% in Quartile 2 to 10.59% in Quartile 4. In contrast, the prevalence among males was relatively higher but did not reach statistical significance (*P* = .5084). Age groups also exhibited variability, with participants aged 31 to 40 showing a notable increase in prevalence from 2.78% in Quartile 2 to 16.30% in Quartile 4 (*P* = .0025). However, older age groups (>50 years) showed less pronounced changes, and these were not statistically significant. Racial analysis indicated no significant differences across groups, with Mexican Americans and Non-Hispanic Whites showing higher kidney stone prevalence in Quartile 4 compared to Quartile 2, but these differences were not statistically significant. Other Hispanic and Non-Hispanic Black populations showed relatively stable prevalence across the quartiles. Education levels did not show statistically significant differences, although there was a trend towards a higher prevalence in Quartile 4 among participants with a high school education or lower. Similarly, marital status and income levels showed trends but lacked statistical significance. Importantly, lifestyle factors like recreational physical activity (*P* = .0205) and smoking history (*P* = .0422) were significantly associated with kidney stone prevalence. Nonsmokers and those with no recreational activity showed higher prevalence in Quartile 4 compared to lower quartiles.

In summary, the subgroup analysis indicates that a higher dietary Ca/P ratio may be associated with increased kidney stone prevalence, particularly in females, younger adults (31–40 years), and those with certain lifestyle factors like smoking and lack of physical activity. These findings suggest that dietary adjustments might be more crucial for specific demographic groups in reducing kidney stone risk.

## 4. Discussion

The prevalence of kidney stone disease is continuously increasing world-widely. Based on a national representative sample data, the present study identified a significant association between dietary Ca/P ratio intake and the risk of kidney stones. The study found that a moderate Ca/P ratio was associated with a reduced risk of kidney stone formation, and the highest quartile of Ca/P intake exhibited a notably higher prevalence of kidney stones compared to those in lower quartiles. Interestingly, this Ca/P ratio served as a better predictor for kidney stone risk than either Ca or P intake alone, highlighting the importance of the balance between these two minerals rather than their individual quantities. This research fills a gap in the literature and suggests that dietary interventions targeting an appropriate Ca/P balance may be more effective for reducing kidney stone risk than strategies focusing solely on Ca or P intake, highlighting the need for personalized dietary recommendations for individuals at higher risk of developing kidney stones.

Various effective therapies are available for kidney stones, and surgical removal strategies is the most commonly used. However, recurrence of the stone remains a big problem for postsurgical management of kidney stone.^[14]^ Thus, the preventive strategies for new and recurrent stone formation should be seriously explored. Among several suggestions proposed by main clinical guidelines,^[2–4]^ dietary management is relatively easy to achieve. Prior investigations revealed that the use of Ca supplements heightened the risk of kidney stone formation.^[15,16]^ High concentrations of Ca and P in urine lead to the supersaturation and subsequent crystallization of calcium phosphate, a key component of kidney stones.^[17]^ Studies show that when calcium phosphate precipitates in the kidney, it lowers urine pH, leading to further crystallization and stone formation.^[^^11,18]^ Maintaining a moderate ratio of Ca to P intake can reduce urinary calcium phosphate saturation, preventing crystal formation. This balance is essential for mitigating calcium phosphate’s role in the development of Randall plaques, which are known to initiate Ca stone formation.^[19]^ Thus, managing Ca and P levels is key to regulating urine chemistry and reducing the risk of kidney stones.

Dietary imbalances, particularly related to Ca and P, can significantly influence the risk of kidney stones. High Ca and P concentrations in urine are major contributors to stone formation, and sodium and animal protein intake increases Ca excretion, further promoting stone formation.^[^^20,21]^ Balanced Ca consumption is protective against stones by reducing intestinal oxalate absorption. However, Ca supplements, if taken outside of meals, may increase urinary Ca excretion without reducing oxalate levels, which increases the risk of stone formation.^[22]^ High P consumption disrupts mineral metabolism, leading to higher kidney stone risk.^[9]^ However, this effect was mitigated when Ca and P were consumed in a balanced ratio.^[9]^ These studies collectively support the importance of maintaining a balanced dietary intake of Ca and P while also managing sodium intake to reduce the risk of kidney stone formation. Few studies utilized the dietary Ca/P ratio to evaluate kidney stone risk and related mineral metabolism. Coltherd et al^[23]^ demonstrated that the Ca/P ratio in diets significantly affects postprandial P and parathyroid hormone concentrations in plasma. They observed that a lower Ca/P ratio led to higher postprandial P levels, which could potentially contribute to kidney stone formation through imbalances in mineral metabolism. However, this study did not further analyze the relationship between Ca/P ratio and the incidence of kidney stones. Another study from Taiwan concluded that the average dietary Ca/P ratio in their population was lower than recommended, which increases the risk of Ca–phosphate imbalances and associated health issues like kidney stones.^[10]^ Likewise, this study did not conduct quantitative analysis to prove the specific relationship between Ca/P ratio and incidence of kidney stones. Our study provides valuable insights into the relationship between the Ca/P ratio and the incidence of kidney stones. It offers specific dietary guidelines regarding the appropriate Ca/P ratio, which are significant for preventing kidney stone formation and ensuring balanced mineral metabolism. The findings contribute meaningfully to the existing body of knowledge and highlight the importance of maintaining a proper dietary Ca/P ratio for kidney stone prevention.

With subgroup analysis, sex, age, activities and smoke may modulate the impact of the Ca/P ratio on kidney stone risk. As is well-known, gender can influence the risk of kidney stones.^[24–27]^ The risk of kidney stone formation is greater in men than in women. Gender-related hormonal and physiological differences likely contribute to this increased risk in men. Estrogen can increase the excretion of citrate inhibiting the formation of certain types of kidney stones, such as calcium oxalate stones.^[24]^ Men tend to have a more lithogenic (stone-forming) urinary profile, characterized by higher levels of substances like Ca, oxalate, and uric acid, which can promote kidney stone formation.^[24]^ Kidney stones affect individuals across a wide age range, with the prevalence and incidence varying among different age groups. The highest prevalence of ever having had a kidney stone was seen in the 40 to 49 age group, with 17.6%.^[28]^ However, the highest incidence of passing a kidney stone within the past 12 months was observed in the 30 to 39 age group, with a rate of 23.6%.^[28]^ While kidney stones have traditionally been more common in older adults, the incidence in younger people, including adolescents and young adults, has been on the rise in recent decades.^[29]^ And younger patients with kidney stones experience a lower health-related quality of life than older patients.^[29]^ The prevalence of kidney stone among young people was more likely influenced by Ca/P ratio in dietary intake. The prevalence of kidney stones is directly related to the level of physical activity, with a dose–response pattern.^[30–32]^ As physical activity increases, the prevalence of kidney stones tends to decrease. With vigorous/moderate recreational activities for at least 10 min continuously per week, dietary intake Ca/P ratio affected kidney stone inconsiderably. Lifestyle and dietary factors, such as fluid intake and sugar-sweetened beverage consumption, also play a role in the gender disparity in kidney stone risk.^[25,33]^ The Dietary Inflammatory Index is a measure that assesses the inflammatory potential of an individual’s diet based on the intake of various nutrients and foods. Liu et al used the Dietary Inflammatory Index to investigate its association with kidney stone formation,^[27]^ finding a statistically significant positive association between a proinflammatory diet, as measured by a higher Dietary Inflammatory Index score, and the risk of kidney stones in females. Similarly, results from the present study showed a change of dietary intake Ca/P ratio influenced kidney stone prevalence in female more significantly than in male. There is a heightened risk of kidney stone development associated with both current smoking and elevated serum cotinine levels.^[34]^ Smoking could impact the prevalence of kidney stone disease through several potential mechanisms, including raising vasopressin levels, which decrease urine output, generating reactive oxygen species that may harm the kidneys, reducing urinary Ca excretion (a protective factor against kidney stones), and elevating urinary levels of metallic elements like cadmium and mercury, which could be related to the formation of urinary tract stones.^[35,36]^ Smokers, with higher intake of dietary Ca/P ratio, were present with higher prevalence of kidney stones according to the finding of present study.

Ca and P in the diet have a significant impact on kidney stone formation. Adequate dietary Ca intake can help bind to oxalate in the digestive tract, reducing the absorption of oxalate into the bloodstream and subsequently lowering the risk of calcium oxalate stone formation.^[22,37]^ On the other hand, excessive dietary P intake, often from high-P foods and drinks, can lead to the formation of calcium phosphate stones in individuals who are predisposed to this type of stone.^[38,39]^ Ca/P ratio of dietary intake seems more appropriate to be an indicator for kidney stone formation management.

Future research should include longitudinal and mechanistic studies, as well as interventional trials, to confirm these findings and explore the underlying biological mechanisms. Based on the findings, we recommend maintaining a moderate dietary Ca/P ratio and tailoring dietary guidelines to individual factors such as age, gender, physical activity, and smoking history to prevent kidney stones. Implementing these recommendations in clinical practice can enhance dietary guidelines and management strategies, leading to more precise and effective interventions for reducing kidney stone incidence and recurrence.

The present work performed comprehensive analysis of the relationship between dietary Ca/P ratios and kidney stone risk, offered practical recommendations, considered different population groups, and contributed to the understanding of dietary strategies for kidney stone prevention. Our study has several limitations, including its observational nature and reliance on self-reported data. Importantly, the initial analysis did not account for accompanying kidney conditions, which could influence the relationship between dietary Ca/P ratio and kidney stone prevalence. We have now addressed this by incorporating accompanying kidney conditions into our logistic regression models. However, further research, including prospective studies and randomized controlled trials, is needed to confirm and expand upon these findings and to provide a more comprehensive understanding of dietary influences on kidney stone formation.

## 5. Conclusion

The findings from this study indicate that a moderate dietary Ca/P ratio is advisable for preventing kidney stones. In subgroup analysis, gender, age, physical activity, and smoking history may modulate the impact of the Ca/P ratio on kidney stone risk.

## Acknowledgments

We appreciate the works by the National Health and Nutrition Examination Survey (NHANES) collaborator.

## Author contributions

**Conceptualization:** Weiyu Zhang.

**Formal analysis:** Benxing Lou, Yu Peng, Feng Wu, Dan Zhang.

**Investigation:** Benxing Lou, Dan Zhang.

**Methodology:** Yu Peng, Feng Wu.

**Resources:** Yu Peng.

**Software:** Dan Zhang.

**Supervision:** Qi Wang.

**Validation:** Benxing Lou.

**Visualization:** Feng Wu.

**Writing – original draft:** Weiyu Zhang.

**Writing – review & editing:** Qi Wang.

## Supplementary Material


