# Supplementary material for: High dietary calcium to phosphorus ratio is associated with high prevalence of kidney stone
Source: Medicine (Baltimore). 2024 Dec 13;103(50):e40778. doi: 10.1097/MD.0000000000040778 (PMC11651432; doi:10.1097/MD.0000000000040778)
Supplement: Supplementary file 1 [file medi-103-e40778-s001.docx]

Supplementary table 1 Data resources in details.

| Item | Resource |
| --- | --- |
| Calcium-to-phosphorus ratio [M (SD)] | Total Nutrient Intakes, First Day/ Second Day |
| Rate of kidney stones (%) | Questionnaire Data: Kidney Conditions - Urology |
| Men (%) | Demographics Data |
| Age [years, M (SD)] | Demographics Data |
| Race (%) | Demographics Data |
| Education (%) | Demographics Data |
| Married (%) | Demographics Data |
| Ratio of family income to poverty (%) | Demographics Data |
| BMI (%) | Examination Data: Body Measures |
| Vigorous/moderate recreational activities for at least 10min continuously per week (%) | Questionnaire Data: Physical Activity |
| Smoked at least 100 cigarettes in life (%) | Questionnaire Data: Smoking - Cigarette Use |
| Daily intake [M (SD)] | Total Nutrient Intakes, First Day/ Second Day |

Supplementary Table 2 Association between intake Calcium and phosphorus and kidney stones

|  | **OR (95% CI), P-value** | | |
| --- | --- | --- | --- |
|  | Model 1 | Model 2 | Model 3 |
| Calcium | 1.0107(0.8071, 1.2656) | 1.0004(0.7942,1.2602) | 1.2796(0.9075,1.8044) |
| *P* value | 0.926 | 0.997 | 0.160 |
| Phosphorus | 0.7948(0.6116,1.0331) | 0.7002(0.5313,0.9228) | 0.5962(0.3351,1.0605) |
| *P* value | 0.086 | 0.011 | 0.078 |

Data are presented as OR (95% CI).

Model 1: crude model.

Model 2: adjusted for age and sex.

Model 3: adjusted for age, gender, BMI, educational level, family income, marital status, race, smoking, vigorous and moderate recreational physical activity, total water drank, energy, alcohol, total intakes (from dietary and supplements) vitamins B6, vitamins C, vitamins D, caffeine, magnesium, sodium, and potassium.

***, *P*<0.001, **, *P*<0.01, *, *P*<0.05.
